# Real-time tracking and detection of patient conditions in the intelligent m-Health monitoring system

**DOI:** 10.3389/fpubh.2022.922718

**Published:** 2022-10-10

**Authors:** Xiaoyan Li, Kangwon You

**Affiliations:** ^1^Department of Physical Education, Jinzhong University, Jinzhong, China; ^2^Department of Physical Education, Jeonju University, Jeonju, South Korea

**Keywords:** health monitoring system, physical activity, cloud computing, Bayesian network, m-Health

## Abstract

In order to help patients monitor their personal health in real time, this paper proposes an intelligent mobile health monitoring system and establishes a corresponding health network to track and process patients' physical activity and other health-related factors in real time. Performance was analyzed. The experimental results show that after comparing the accuracy, delay time, error range, efficiency, and energy utilization of Im-HMS and existing UCD systems, it is found that the accuracy of Im-HMS is mostly between 98 and 100%, while the accuracy of UCD is mostly between 98 and 100%. Most of the systems are between 91 and 97%; in terms of delay comparison, the delay of the Im-HMS system is between 18 and 39 ms, which is far lower than the lowest value of the UCD system of 84 ms, and the Im-HMS is significantly better than the existing UCD system; the error range of Im-HMS is mainly between 0.2 and 1.4, while the error range of UCD system is mainly between −2 and 14; and in terms of efficiency and energy utilization, Im-HMS values are higher than those of UCD system. In general, the Im-HMS system proposed in this study is more accurate than UCD system and has lower delay, smaller error, and higher efficiency, and energy utilization is more efficient than UCD system, which is of great significance for mobile health monitoring in practical applications.

## Introduction to physical activity monitoring

Numerous studies have shown that multi-channel environmental monitoring systems can be used to observe human physical activity, thereby assisting and analyzing individual healthcare functions (1). Creating and implementing a precise interconnected medical infrastructure can predict individual health problems using the rapid medical system in psychiatric emergency services (PES) (2, 3). The medical field can integrate medical innovation, artificial learning, smart IoT, and other advanced future technologies to facilitate the creation of wearable Internet of things (IoT) as medical devices (4–6). The system detects the physical signs of an individual's body and notifies doctors as early as possible in the event of a serious illness. The computer includes wireless chips for data collection, battery energy recovery, and data management levels (7).

Existing applications in the smart healthcare industry are run to monitor physical activity in a multi-access physical control system in a cloud network (8). Cloud computing technology (CCT) facilitates the transmission of health information collected and analyzed by IoT devices through the Internet through different deep learning architectures, machine learning and convolutional neural networks, implemented in the cloud (9, 10). The current era of multi-access visual surveillance schemes has seen a huge boom in physical surveillance (11), which lists the main issues for this generation of investigators in qualifying situations and data processing:

- Big data collection can be streamed. The continuous production of large cloud-based datasets results in massive data collection. Heterogeneous datasets (12) have more potential errors.
- Incorrect timing relationships of IoT devices can lead to noisy data, failures, incorrect data transmission to physical surveillance networks with multiple access rights, and higher-cost and higher-capacity system bottlenecks (13, 14).

These features increase the complexity of wearable IoT devices, with fluctuations in computational variables such as latency, accuracy, performance, standard error, and more power consumption (15). This inconsistent user response between cloud and IoT leads to network problems with a large number of I/O issues to provide stable health information for physical monitoring systems with multiple access rights (16). Current IoT approaches have less reaction time because of interrupted and discrete data transmission, with long gaps in information gathering (17, 18).

This approach can be a more comprehensive and promising solution to the current challenges of the multi-layered physical observation and fitness monitoring market in personal physical activity. Contributions to this article are as follows:

- A new network of neutral, streamlined, tightly interconnected layers for determining healthy imbalances in heat,
- A Bayesian machine learning system embedded in a wearable IoT smart data analysis patch for predicting organ dysfunction,
- A complete physical monitoring infrastructure using cloud technology, interactive technology, real-time analysis of IoT information using evolutionary training,
- An optimized model for classification of different human physical activity recognition using Bayesian neural cloud technology.

Based on this, this paper designs and implements an intelligent mobile health monitoring system (Im-HMS) after investigating the background and literature about physical activity monitoring systems and analyzes and evaluates its performance, aiming to improve the performance of mobile health monitoring systems through mobile health networks to monitor human physical activity in real time, thereby reducing health risks (19, 20).

The innovation of the research lies in the combination of cloud computing, the Internet of things, artificial intelligence, etc., and creatively designed a breakthrough mobile health network, so as to monitor the health of patients in real time and provide important information for patients to pay attention to their own health and reduce the risk of pathology tool.

## Background of physical activity monitoring system

Edge and cloud technologies are the best tools for analyzing data sources in different healthcare systems (21). Mobile cloud computing is often used as the state of the art in the modern practice of physical surveillance devices with multiple access rights. Although these techniques are touted for their encouraging performance, the latency and accuracy of transferring large health datasets across the system are significant for these techniques (22). In terms of efficiency and energy usage, the use of neural network statistical computations when analyzing health datasets does not yield efficient performance. Clinical research on external multi-access control technologies is significant, mainly focusing on reducing people's health risks (23). Some findings suggest that telemedicine services can be used anywhere, but data-driven approaches are being used to diagnose already multifaceted anatomical improvements (24).

This method can achieve a reliability rate of 82%, making it unsuitable for individual health studies with higher precision and difficulty (25). A group of academics from Columbia University used recurrent network approximation and convolutional neural architecture (CNA) to study the body motion and heart rhythm of individuals, and they achieved excellent performance (26). Intensive data analysis (IDA) has recently been used to include local critical health monitoring with greater power and significant error rates.

Mobile health (m-Health) initiatives, including text messaging, have been successful in improving blood sugar management for heart disease and regular physical activity (27). Poorer people, such as those with low incomes, diabetes, and depression, who are geographically and culturally diverse, have additional barriers to daily physical activity. With the development of mobile health strategies targeting depressive symptoms, understanding the needs and barriers to increased physical activity among disadvantaged groups is critical for greater end-client engagement (28, 29).

In several recent surveys, with regard to lower information technology and health status, smartphone ownership in this population has shown an increased level (93%) (30). Despite the effective development of online interventions to improve physical activity in a variety of clients, there has been little publicity about the developmental process of initiatives such as the introduction or use of clinical practice. It shows that it is not satisfactory to include the end user's needs before the developmental process. Successful approaches combine information adaptation to provide tailored intervention procedures and direct input from various end clients (31).

It uses user-centered decoding (UCD) to propose content and interaction requirements in designing text-based physical activity interaction, diabetes, and mental health response alert monitoring and assessment experiments to recognize the importance of including consumers in the early stages of developmental sexual process. The program attempts to use an interface to gather steps and a framework to teach people to customize information so that clients with diabetes and anxiety disorders can increase physical activity (32, 33).

The theoretical structure of UCD consists of an established method of centrally interpreting the identity of the end user (the patient) in order to make m-Health treatments relevant and usable to the target group (34). This paper recognizes common challenges and enables France and Italy to talk about obesity, comorbid anxiety, and physical activity reduction through text messages and physical activity (35, 36).

In recent years, condition-based monitoring (CBM) has been used to rapidly identify defects and other suspicious behaviors in the body to track different signals of the human body in multi-access physical monitoring systems. It does not contain details of human wellbeing due to excessively strong binding (37). Due to the dynamic nature of this method, it is not suitable for diagnosis (38). One of the potential solutions to the multi-access physical tracking device problem is the advancement of artificial learning models and regressions for classifying health data sources or physical activity content (39). A fully automated mobile health intervention with tracking and texting components increases physical activity (40). SMS intervention can help people make changes in behavior such as exercise, but little is known about how this intervention works and what factors affect people's responses (41). At the same time, certain attractive landscapes and facilities can enable children to increase physical activity on campus (42, 43).

Through the research of domestic and foreign scholars, it can be found that IoT technology has been implemented and combined with wearable IoT devices to analyze human pain through optical surface electromyography. Based on this, the reliability of the preparation conditions can be reduced by the method outlined in this paper, and at the same time, the smart plug-and-play system IoT solves the problem of energy usage. Therefore, it is of practical significance to study the automatic tracking problem in the intelligent detection system in this paper (44).

## Proposed intelligent mobile health monitoring system

As one of the key application areas of pervasive computing is effective health management, more advancements are needed in mobile technology in healthcare. This integration improves communication between patients, physicians, and other healthcare professionals. It allows accurate medical information to be transmitted from any location *via* mobile devices. Advances in sensors, low-power integrated circuits, and wireless networks have made it possible to build low-cost, tiny, lightweight, and intelligent biosensor nodes. This section discusses an intelligent mobile health monitoring system (Im-HMS), which uses biomedical and environmental data collected by deployed sensors to provide medical input to patients *via* mobile devices.

**Figure 1:**
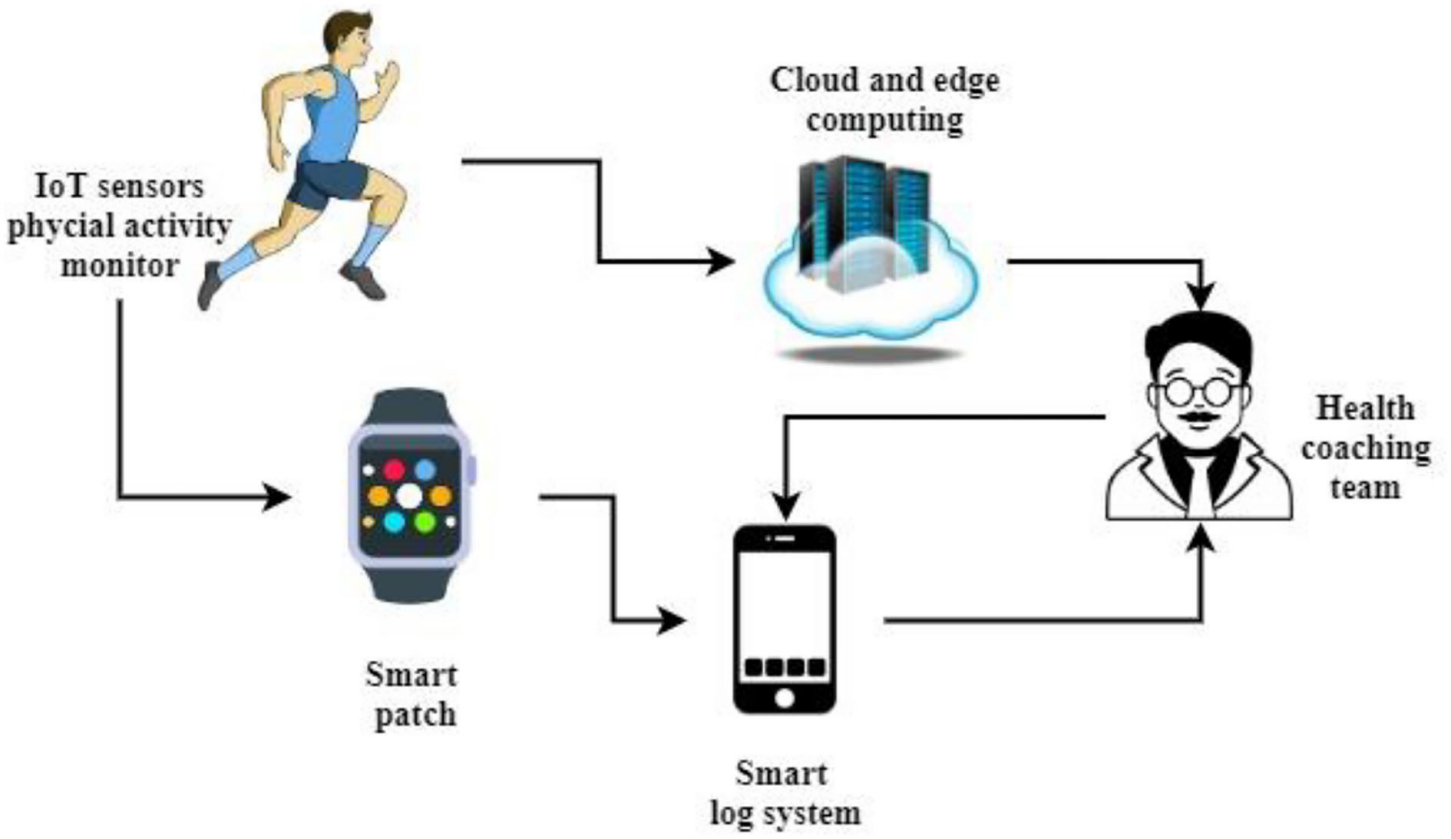
The architecture of the proposed intelligent m-Health monitoring system (Im-HMS).

Figure 1 shows the architecture of the proposed intelligent mobile health monitoring system (Im-HMS). IoT sensors monitor the user's physical activity and send it to a cloud computing database, and the Fitbit patch also tracks the user's heartbeat. It is sent to the mobile device when the system is logged in; based on the IoT sensor information and the intelligent log system, the m-Health information is sent to the health coach system for detailed analysis of complex issues; otherwise, the automated system will send SMS to the user. Cloud computing uses a hybrid cloud and network framework to solve large-scale physical dataset management challenges. Health-related data collection can be stored on an edge computing network consisting of a layer of specific IoT sensor monitoring units, the edge, and an intelligent logging system that intelligently processes IoT data using human cognitive systems. Edge networks for accurate diagnosis and prediction of body trends have been combined with multiple sensors such as blood, heat, EMG, ECG, EEG, pressure, vision, respiration, accelerometers, and cluster heads.

Advanced computing technology captures data where information is required using decentralized devices. This wearable smart logging board with an IoT monitor in a cloud computing environment provides reliable and practical information about an individual's physical movements for physical observation of children and adults in a multi-access physical monitoring system. Behind the scenes, the tracked information is analyzed in two different ways:

- Diagnostic specialist,
- Online cloud backup.

Early detection of organ dysfunction is more nuanced and takes longer to report. It has been done with edge frames. It uses intelligent gateways, computing units, and powerful remote network resources and is more suitable for physical surveillance of individuals through multi-access tracking devices. A mobile wearable mat with IoT sensors communicates large datasets with the framework *via* local networks including Wi-Fi and NFC.

In this technique, a Bayesian learning network is used to accurately track the physical movements of individuals in a decentralized system at the edge of the computing world. The first process of the service is to assess and isolate characteristics or patterns of healthcare information sets. The processed normalized dataset minimizes data accessibility and duplication. The framework includes a layer of data, polyphase or hidden units, and an input module.

These frameworks are built into Bayesian networks, where matrices consist of different IoT databases represented in the form of inputs that are converted numerically into vectors. Each level has the same connection to the cross-level defined as linear regression. This regression is analogous to the fluctuations of a small part of the human mind. The values obtained from this normalization operation are “mean (μ) and standard deviation (μ).”

Case: 1 mean = 0, mean deviation = 1

The smart log patch for wearable sensors displays a network entry *y* = {*y*_1_, *y*_2_, *y*_3_, ⋯ , *y*_*i*_}, where the *i* = {1, 2, ⋯ , *N*} input level is analyzed through the IoT device's full *N*range of data sources and transferred to a filter layer to reduce noise by evaluating the input matrix number. There are some depolarization and repolarization trends in the data source, in which *N*_*i*_ are analyzed using mean and default values, as shown in Equation (1),


(1) $$\begin{array}{l} Ni=\frac{yi-ȳi}{\sigma },f\left(Ni\right)=\{\begin{array}{c} Ni,i<N\\ Ni,else \end{array} \end{array}$$

where *N*_*i*_ represents the standardized input dataset used in the machine learning framework. *f*(*x*) varies in a different range from 0 to *N*. *y*_*i*_ represents the input to the system, and the average value of the input is denoted as ȳ_*i*_. The standard deviation of the input is expressed as σ , and the study uses gradient descent to make the same update of the parameters.

**Figure 2:**
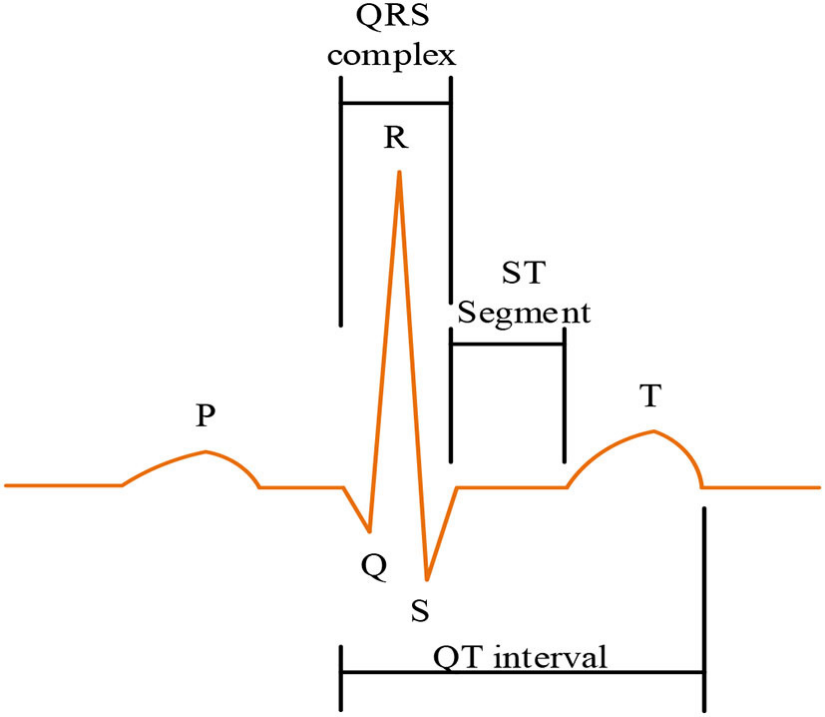
Illustration of ECG signal.

Figure 2 shows a graphical representation of the ECG signal. It facilitates extraction of electrocardiogram (ECG) traces, which include *P*atrial hyperpolarization waves, denoted as *T*, and ventricular hyperpolarization waves, denoted as. During this procedure, the structure of the brachial plexus (*U*) is often ignored as it is not usually shown. The four objects are evaluated for accuracy from source to target through a Bayesian network. In the form of null and large logic, the mean ( ȳ_*i*_) and standard deviation ( σ ) are represented by Equations (2) and (3), respectively. It is worth noting that in order not to ignore some important indicators and reduce errors, which will affect the results of data analysis, the study here normalizes the mean and standard deviation.


(2) $$\begin{array}{l} {ȳ}_{i}=\frac{\sum_{i=0}^{N}y_{i}}{N}f\left(N_{i}\right) \end{array}$$

(3) $$\begin{array}{l} \sigma =\sqrt{\frac{y_{i}-{ȳ}_{i}}{N-1}} \end{array}$$

Equations (2) and (3) support the data definition and normalization spectrum from the extracted feature layer analysis of all health data sources. *N*_*i*_ represents a collection of inputs accessed during the learning process. *f*(*x*) varies from 0 to N, which is a function of the input. *y*_*i*_ is the output of the first stage of the framework, and the average is reported as ȳ_*i*_. The normalized deviation value is given as σ.

Case: Intelligent log device routing and data collection

**Figure 3:**
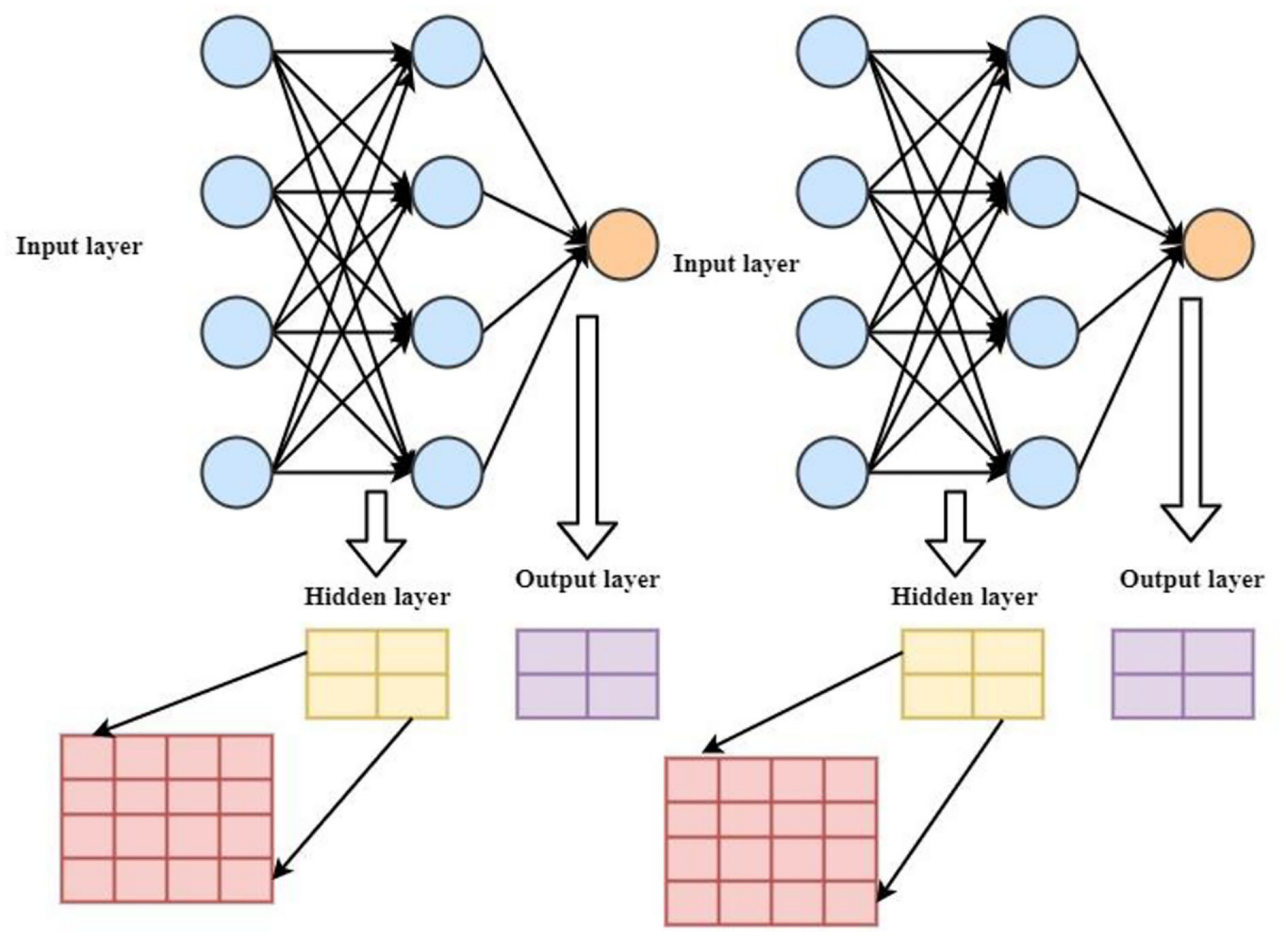
Systematic model of the proposed intelligent m-Health monitoring system (Im-HMS).

Figure 3 shows the system model of the proposed intelligent mobile health monitoring system (Im-HMS). It contains three layers, namely, input layer, output layer, and hidden layer. In the input layer, on the one hand, the output to the hidden layer is crossed with each other, and on the other hand, the input and output layers are merged, wherein the amplifier, cache, and static random access memory (SRAM) setting unit are IoT data processing structures. It is used to control the output data by using data selection, “multiplexer” method, and data collection detectors as different sensors to keep equipment archives. Both P-MOS and N-MOS have three modes including adaptive, sleep, and snooze. In this case, summary statistics and *F*(*IG*)data benefit ratios were analyzed using large health datasets. The income is shown in Equation (4):


(4) $$\begin{array}{l} F\left(IG\right)=IG_{i}-\overset{N_{i}}{\underset{i=1}{\sum }}\frac{NI\left(y_{i}\right)}{NI}F\left(IG_{i}\right) \end{array}$$

where $\overset{N_{i}}{\underset{i=1}{\sum }}\frac{NI\left(y_{i}\right)}{NI}$ means that the specific *NI* number varies between 1 and *NI*a *i*set of data and F(IG) represents the data benefit ratio, which is analyzed using a large health dataset. The information of the smart sensor is computed on the hidden unit using wear values that depend on different iteration stages, and the filtering layer helps to denoise and average the marginal error. To determine the estimation accuracy of the sensor results, the time-out ratio was calculated using the gain ratio (GR), which is the ratio of and its log variables *logh* (*i*) shown in Equation (5).


(5) $$\begin{array}{l} GR=\frac{F\left(IG\right)}{\sum_{i=1}^{N_{i}}h\left(i\right)logh\;\left(i\right)} \end{array}$$

where *h*(*i*) is the parts of the hidden layer and filter layer database *i*vs. *i* = {1, 2, ⋯ , *N*}, and time-varying ratios were calculated using the gain ratios (*GRs*), which are the ratios of and their log variables *logh* (*i*). Proxy and level of developmental mode I device routing logic multiplication are shown in Equations (6) and (7).


(6) $$\begin{array}{l} H{\left(L\right)}_{N}=\mu_{1}\left(y_{1}\right)\vee \mu_{2}\left(y_{2}\right)\vee \cdots \mu_{3}\left(y_{3}\right)\vee \mu_{N}\left(y_{i}\right) \end{array}$$

(7) $$\begin{array}{l} H{\left(L\right)}_{N}=\mu_{1}\left(y_{1}\right)*\mu_{2}\left(y_{2}\right)*\cdots \mu_{3}\left(y_{3}\right)*\mu_{N}\left(y_{i}\right) \end{array}$$

where is the minimal logical process. The ∨ filter function is expressed as μ_*i*_ (*y*_*i*_), and *i*varies from *i* = {1, 2, ⋯ , *N*}. In the IoT architecture, the data management nature of the transition is used and done with a tri-mode shutter that retains information during the snooze phase. The three-mode switch is built on unique low-power IoT architectural approaches such as data retention power limiters, multiple pause stages, and on-chip acceleration for dynamic power.

Case: 3 systems programming for Bayesian learning

It implements agile learning because it creates a balance between the information recorded in terms of difficulty or distraction and the consistency of the extracted feature layers. That is because, especially in real-world environments, detecting multi-access physical monitoring applications' health data on cutting-edge systems and large databases is more complex. Data are normalized to prevent transmission and accumulation of data, as shown in Equations (8) and (9):


(8) $$\begin{array}{l} K^{t}=y^{k}-F{ȳ}^{k} \end{array}$$

(9) $$\begin{array}{l} D_{N}=\frac{F\left(K^{t}\right)}{\sqrt{var\;\left(y^{k}-F{ȳ}^{k}\right)}} \end{array}$$

*y*^*k*^ − *Fȳ*^*k*^ is the variance between the kth input measurement and the standard data source. *D*_*N*_ is the sample variance ratio of the kth-level database. varies in a different range from 0 to N. It is mainly used to minimize the unintended noise floor of smart logging devices due to external frequencies. The computational difficulty of smart log patches is minimized by adaptive triggering of training layers. The key elements of the neural classifier are represented by Equations (10)–(13):


(10) $$\begin{array}{l} x^{k}=\propto^{k}y^{k}-\beta^{k}Y \end{array}$$

(11) $$\begin{array}{l} \propto^{k}y^{k}=N_{R1} \end{array}$$

(12) $$\begin{array}{l} \beta^{k}Y=N_{R2} \end{array}$$

(13) $$\begin{array}{l} x^{k}=N_{R1}-N_{R2} \end{array}$$

where *N*_*R*1_ − *N*_*R*2_ is the disturbance factor generated by ∝^*k*^*y*^*k*^ and β^*k*^*Y*, in which ∝^*k*^*y*^*k*^ and β^*k*^*Y* are the input variables of the agility training linear kernel function. They can have a noiseless computing system *x*^*k*^ (in this trigger function, a probability distribution factor from the source layer to the target layer is added, improving the prediction accuracy by preserving the bad switching activity inside the network throughout the operation of the smart log board, and contributes to energy saving). The Gaussian finite activation mechanism is represented by Equations (14) and (15):


(14) $$\begin{array}{l} F\left(g,h\;|\;\varphi \right)=\overset{N}{\underset{i=1}{\sum }}\frac{v_{n}-h_{n}}{var\;\left(\sigma_{i}^{2}\right)}-\overset{k}{\underset{i=1}{\sum }}\frac{W_{n}*h_{n}}{\sigma_{i}^{2}}*\overset{N}{\underset{i=1}{\sum }}\frac{v_{n}}{\frac{1}{\sigma_{i}^{2}}}\\ \;-\overset{N}{\underset{i=1}{\sum }}\frac{v_{n}}{\sigma_{i}^{2}} \end{array}$$

(15) $$\begin{array}{l} F\left(g,h\;|\;\varphi \right)=\overset{N}{\underset{i=1}{\sum }}\frac{v_{n}-h_{n}}{var\;\left(\sigma_{i}^{2}\right)}-\overset{k}{\underset{i=1}{\sum }}\frac{W_{n}*h_{n}}{\sigma_{i}}v_{n}\\ \;-\overset{N}{\underset{i=1}{\sum }}\frac{v_{n}}{\sigma_{i}^{2}} \end{array}$$

where *F*(*g, h* | φ) is a finite function of the Gaussian stimulus; *v*_*n*_ is the apparent receptor; *h*_*n*_ is the invisible receptor; σ_*i*_ is the standard error which is a finite function of the logarithmic stimulus; and *W*_*n*_ is the neuron weight. Information difficulty with a time factor in minutes is calculated using machine learning methods in the scalable training of neural networks *n*/*m*. Here, *y* is an overall data database of fixed length and time information T estimate a given input, *y*, which *n*/*m* can reduce the difficulty if it raises platform constraints *T*.

The Im-HMS framework is designed on an accessible, service-oriented infrastructure using portable sensor technology and a web application programming interface (API) to allow service users and cardiac rehabilitation (CR) professionals to messaging for accurate data interaction—in real time. The following is a detailed description of the key elements and overall design of the Im-HMS framework.

### Architecture design

Im-HMS consists of (a) a wireless Fitbit heart rate monitoring device, (b) an Android, iOS, or macOS device, (c) a mobile computer and cloud-based portal Fitbit, (d) an online therapy platform, and (e) instant messaging *via* SMS. Fitbit was selected for its wearable activity monitoring system because it is very popular, effective, affordable, and user-friendly. Fitbit is the leading device in the wireless health and wellness market and has historically been the top economy in the United Nations.

In various validation trials, Fitbit's activity monitor has been accurately checked during exercise, screen time, rest, and pulse rate measurements. Their effectiveness, availability, and appropriateness have been demonstrated in several intervention trials affecting various populations and age categories, including coronary heart disease (CHD) patients participating in CR services. Smart devices have been seen as the ideal medium for connectivity. Mobile Internet access enables remote control of intense fitness and fitness trainers in nearly every area *via* text message.

Fitbit Galaxy Gear, Surge 2, and Spike use 3D and heart rate HR optical activity trackers to record sedentary time, sleep time in hours, and calorie intake, including strength-related minutes. Although Im-HMS is flexible and can capture user activity from other systems, compared with other activity metrics, Im-HMS is beneficial for tracking individuals with CHD, resulting in more accurate measurements of sustained HR and caloric expenditure.

Fitbit gadgets integrate with the Fitbit smartphone app *via* Bluetooth Low Energy (BLE) network technology, allowing users to identify a view of their daily metrics by wirelessly importing operational data into the app. Fitbit's smartphone app offers a range of capabilities and changing resources, including orientation, automatic monitoring of activity habits and goals (physical activity (PA), fitness, and screen time), success reviews, inspiration/questions, social security, bonuses, and more.

Synced data are immediately forwarded from the Fitbit smartphone app to the Fitbit cloud server every 15–25 min or when the app is accessible to individual users. When CR respondents upload information from their Fitbit device to the server, the Im-HMS database is immediately notified *via* Fitbit's registration API. It enables Im-HMS to obtain current data on respondents without implementing sampling or planning schemes. Im-HMS starts some API calls to get real information from Fitbit cloud storage service and saves them securely in its repository (motion, active time, workout, heart, and sleep).

**Figure 4:**
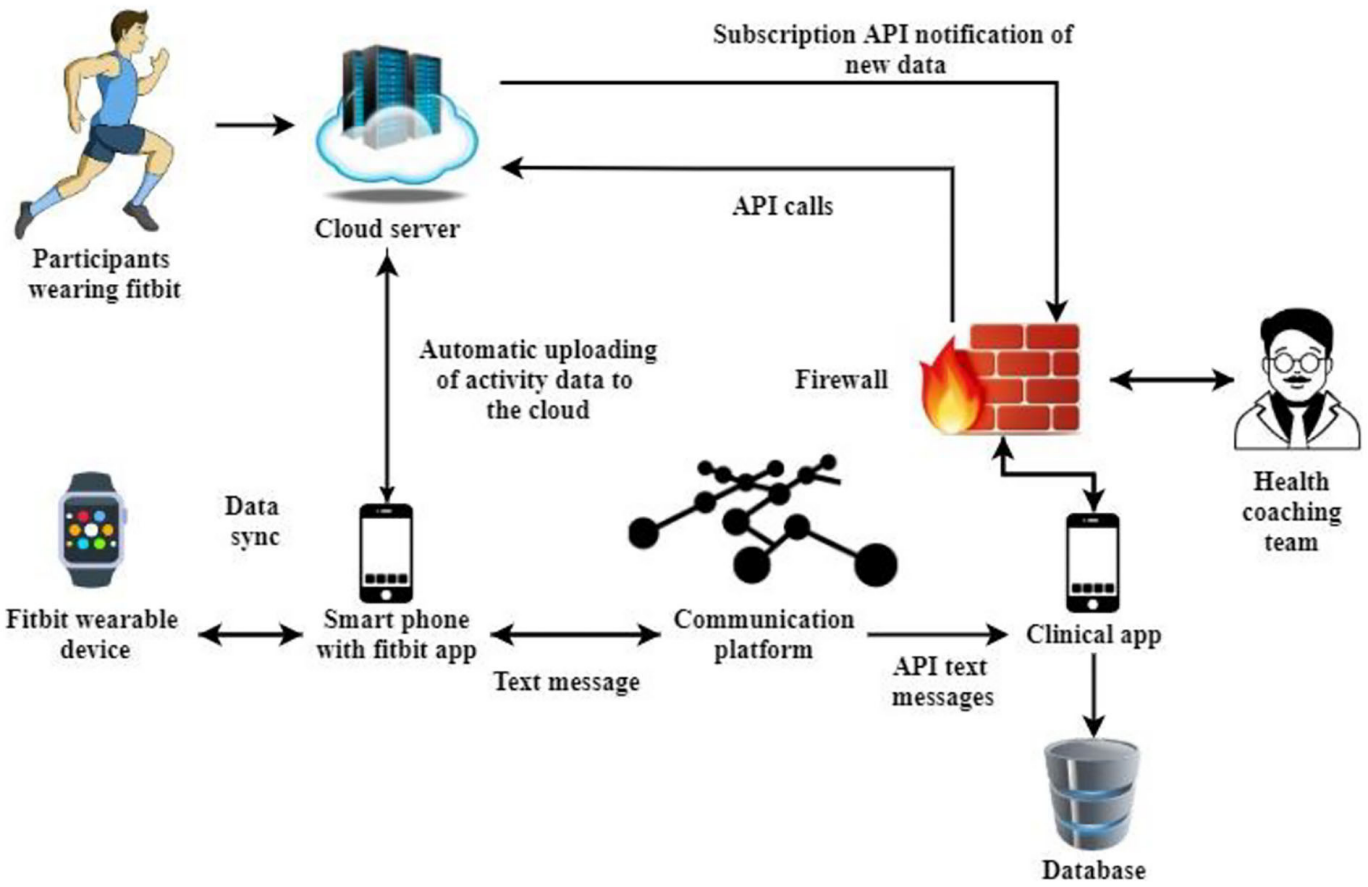
Cloud interface of the proposed intelligent mobile health monitoring system (Im-HMS).

Figure 4 shows the cloud interface of the proposed intelligent mobile health monitoring system (Im-HMS). Im-HMS's medical framework (screen) is an applicable web-based password and gateway. The Im-HMS platform provides a user-friendly interface where CR professionals monitor the activity details of each client and send motivational text messages to mobile phones *via* SMS to increase PA and reduce sedentary behavior. The text messaging platform comes with a therapy dashboard that uses the Twilio API to deliver texts and emails to clients with recorded CRs. Twilio is a cloud-based messaging network that allows application programmers to dynamically transmit text messages through its web services API. Text messages submitted from the Im-HMS can be repeated (e.g., daily) or sent to one or even multiple respondents at a specified date and time.

### Regular exercise and activity remote

The main elements of a CR system must assess daily activities and health status and other significant risks. The latest Fitbit fitness tracker captures body and HR pace, length, and strength in real time. They realized the rapid detection and recording of sustained high-activity movement and enhanced fitness, such as walking, running, or cycling, through a revolutionary feature called “SmartTrack.”

Im-HMS collects time-series information related to PA and HR from the Fitbook cloud platform in proximity and visualization tools that provide meaningful, interactive views of the data to aid in effective remote control and provide CR patients and each location setting goals. Using the calendar feature, CR professionals can display minutes and HR details for each client, including totals measured, intensity-rated PA minutes, and calories burned.

Compared with intraday information, Im-HMS facilitates monitoring and visualization of PA data over long periods of time. In terms of physical activity and exercise, CR professionals can select a date set to see each individual's improvements and patterns over time. These steps are complemented by a map showing the Fitbit's current wearing period. Im-HMS uses new technology to use Fitbit's heart rate monitor to correctly measure the cumulative minutes each person uses on any given day.

This calculation of device wear time serves the following three functions: (a) if the Fitbit device is not in use, automatically send a text message to the customer, (b) assess whether the Fitbit user's decline in exercise is due to an improvement in PA behavior, and (c) collect information to organize time without sufficient data. The framework is designed for automatic text message transmission and sequencing.

### Remote time and behavior reporting

Numerous observational trials and meta-analyses have shown that coronary-specific and total mortality are associated with physical inactivity. Sedentary behavior was associated with any sleep behavior marked by energy expenditure in a sitting or resting position. The adverse effects of prolonged sitting have been reduced. Im-HMS uses software to (a) record and visualize each person's screen behavior and (b) automatically send personalized text message alerts when inactivity is determined to enable customers to reduce their physical activity behavior.

### Adjust SMS operation

Cell phone texting is a useful weapon for changing behavior because it is readily available, inexpensive, and easy to use. Extensive literature spanning years of research shows the beneficial effects of texting on health status and behavior. Texting should be used frequently to foster PA and other positive habits in CHD patients by providing elements of instruction, prevention, and self-digestion based on behavioral theoretical frameworks and techniques.

Im-HMS is designed to help create, tag, store, and distribute user and community text messages. CR professionals can use Im-HMS to develop and use the identification and labeling resources in the framework for easy access and recovery in a practical way. Arriving Fitbit results and each individual's progress can be adjusted based on the nature of the text message. Text can be delivered immediately, repeatedly, or at a scheduled time stamp. In addition, Im-HMS encourages the receipt of texts from CR patients to enable a two-way connection between patients and clinicians.

## Software analysis and performance evaluation

In this paper, different health databases are linked by placing a smart wearable scoreboard to assess the different behaviors of neurons throughout the body. It supports monitoring of human cognitive processes through palm and heel pressure, visible gyroscopes, breathing gyroscopes, and EMG, ECG, and EEG.

Table 1 shows the simulation parameters of the proposed intelligent mobile health monitoring system (Im-HMS): IoT device power supply 0.9 V, chip size 6 mm, clock frequency 100 MHz, 16 ports, and 14 sensors, using Bluetooth and Wi-Fi technology.

**Table 1:** Simulation parameters of intelligent mobile health monitoring system (Im-HMS).

**Parameter**	**Value**
Power	0.9 V
Chip size	6 mm
Clock frequency	100 MHz
Input–output port	16
Sensors	14

**Figure 5:**
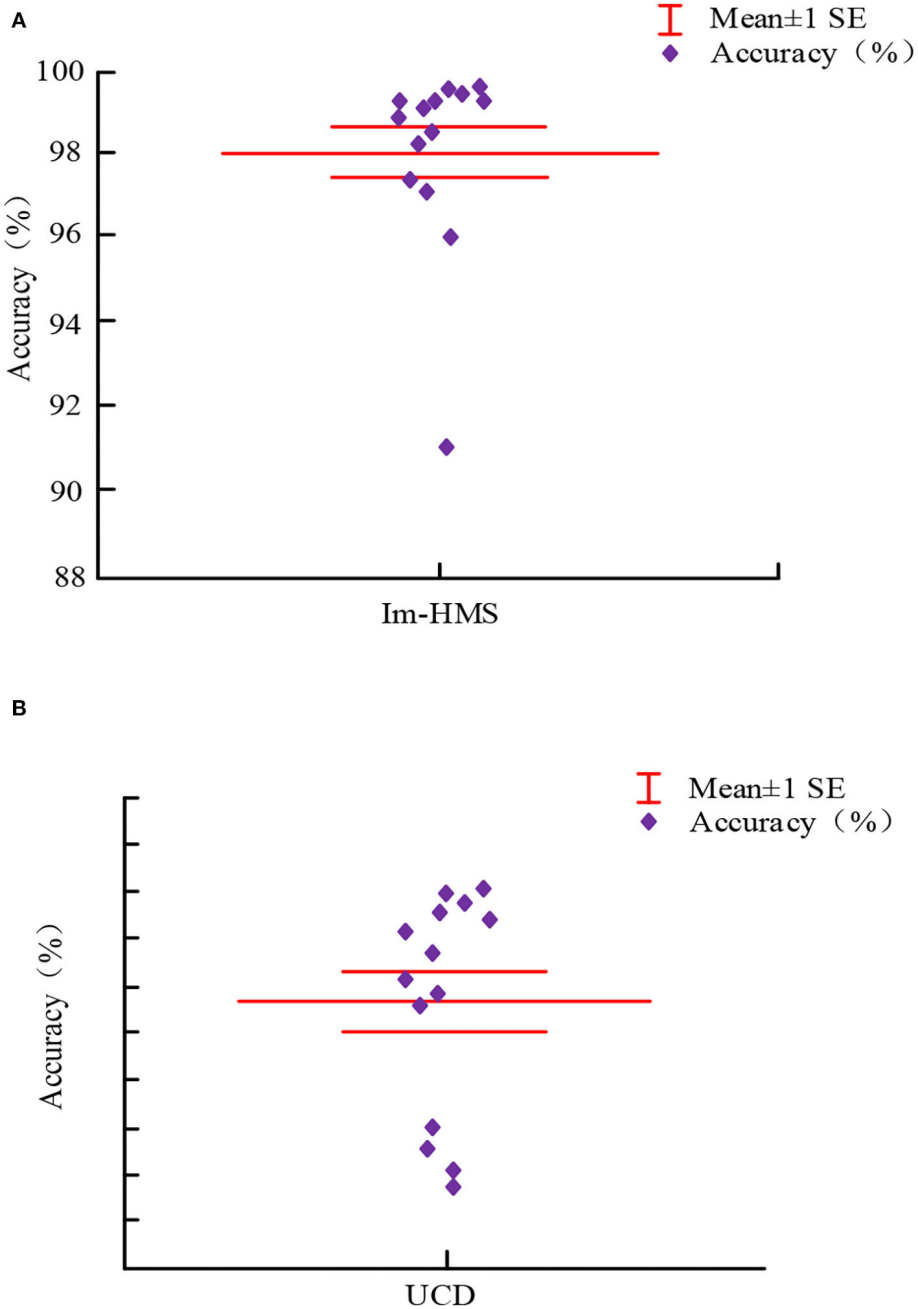
Accuracy comparison between Im-HMS system and UCD system. **(A)** Accuracy analysis of the proposed Intelligent m-Health monitoring system (Im-HMS). **(B)** Accuracy analysis of the existing UCD system.

Figures 5A,B shows the accuracy analysis of the proposed intelligent mobile health monitoring system (Im-HMS) and the existing UCD system, respectively. A total of 14 sensors were considered for simulation analysis. The accuracy of the proposed intelligent mobile health monitoring system (Im-HMS) and existing systems is analyzed and plotted in the figure. The results show that, among all 14 IoT devices, the accuracy of Im-HMS is in the range of 91–100% overall, most of them being in the range of 98–100%, while the overall accuracy of the UCD system is in the range of 83–97%, most of them being between 91 and 97%, so the accuracy of the intelligent mobile health monitoring system (Im-HMS) proposed in this paper is higher than that of the existing UCD system.

Table 2 shows the latency analysis of the proposed intelligent mobile health monitoring system (Im-HMS). The transmission of IoT devices ranges from a minimum of 10–100% for simulation analysis. The end-to-end latencies of the proposed system and the existing UCD system are calculated and given in the table. The results show that the proposed intelligent mobile health monitoring system (Im-HMS) has the lowest end-to-end latency among all IoT devices compared with existing systems.

**Table 2:** Latency analysis of the proposed intelligent mobile health monitoring system (Im-HMS).

**Transmission range (%)**	**Im-HMS(ms)**	**UCD (ms)**
10	39	88
20	37	89
30	32	91
40	29	92
50	27	89
60	24	86
70	23	84
80	21	88
90	19	90
100	18	92

**Figure 6:**
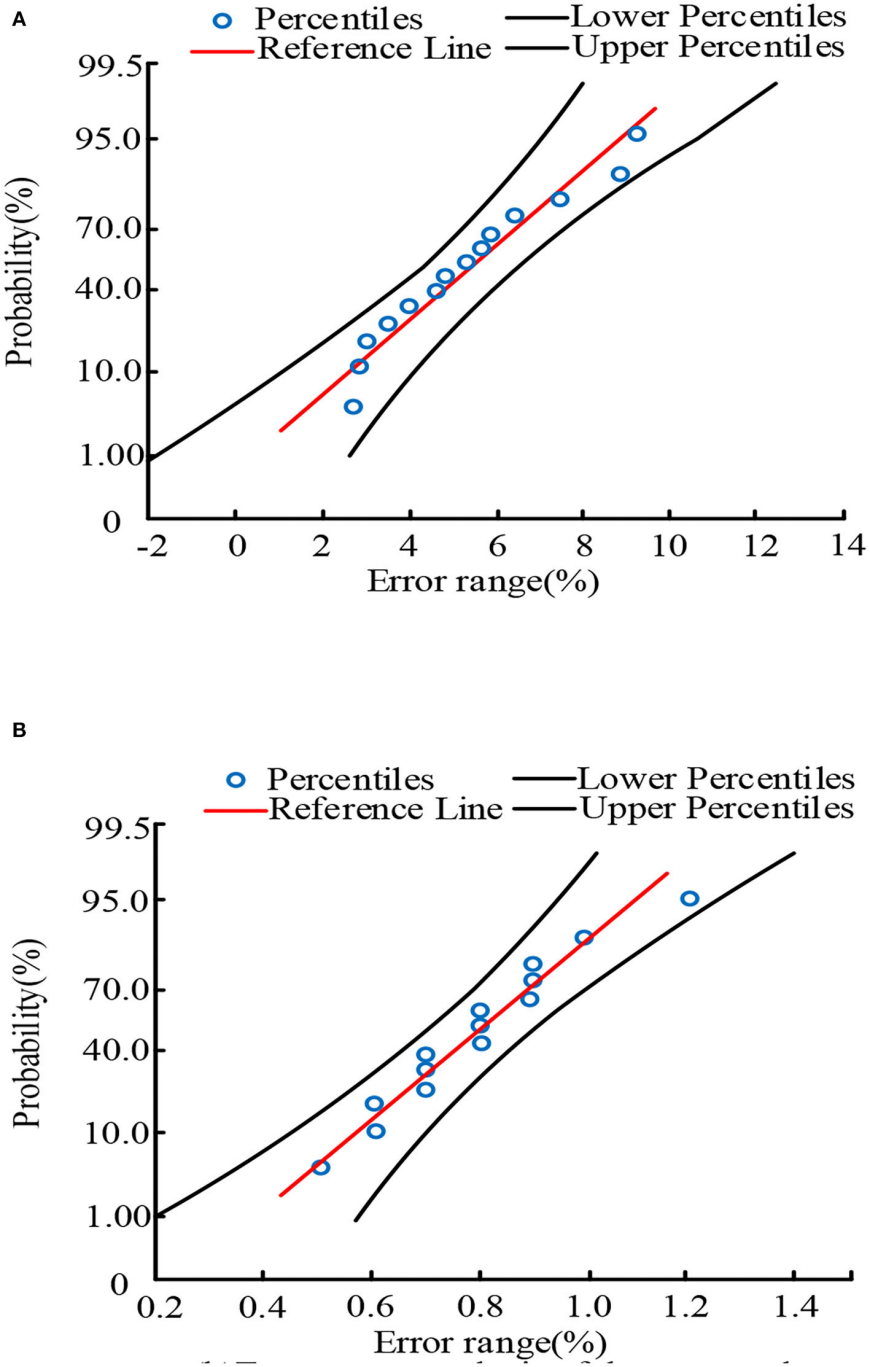
Error comparison between Im-HMS system and UCD system. **(A)** Error range analysis of the existing UCD system. **(B)** Error range analysis of the proposed Intelligent m-Health monitoring system (Im-HMS).

Figures 6A,B shows the error range analysis of the existing UCD system and the proposed intelligent mobile health monitoring system (Im-HMS), respectively. In Figure 6A, the mu value is set to 5.30714 and the sigma value is 2.12511; in Figure 6B, the mu value is set to 0.79286 and the sigma value is 0.1872. The error bounds of the proposed intelligent mobile health monitoring system (Im-HMS) and measured against existing systems are shown in the figure. As can be seen from the figure, the error range of Im-HMS is between 0.2 and 1.4, while that of UCD system is between −2 and 14. The Im-HMS system has significantly smaller error than UCD system, and its output shows that the proposed intelligent mobile health monitoring system (Im-HMS) has the lowest error among all sensor nodes. The mean and deviation values of the system are shown in the figure.

Table 3 shows the efficiency analysis of the proposed intelligent mobile health monitoring system (Im-HMS). The efficiency of the system is calculated as the ratio of output to input. The efficiency of the proposed framework and existing models is evaluated and given in the table. This finding shows that the proposed intelligent mobile health monitoring system (Im-HMS) has the highest efficiency among all sensor nodes.

**Table 3:** Efficiency analysis of the proposed intelligent mobile health monitoring system (Im-HMS).

**IoT sensor**	**Im-HMS (%)**	**UCD (%)**
1	91	88.7
2	97.2	89.2
3	97.4	91.3
4	97.8	92.7
5	98.1	93.2
6	98.3	93.8
7	98.5	94.1
8	98.6	94.8
9	98.7	95.1
10	98.8	95.6
11	98.9	95.8
12	99.1	96.1
13	99.2	96.3
14	99.3	96.4

**Figure 7:**
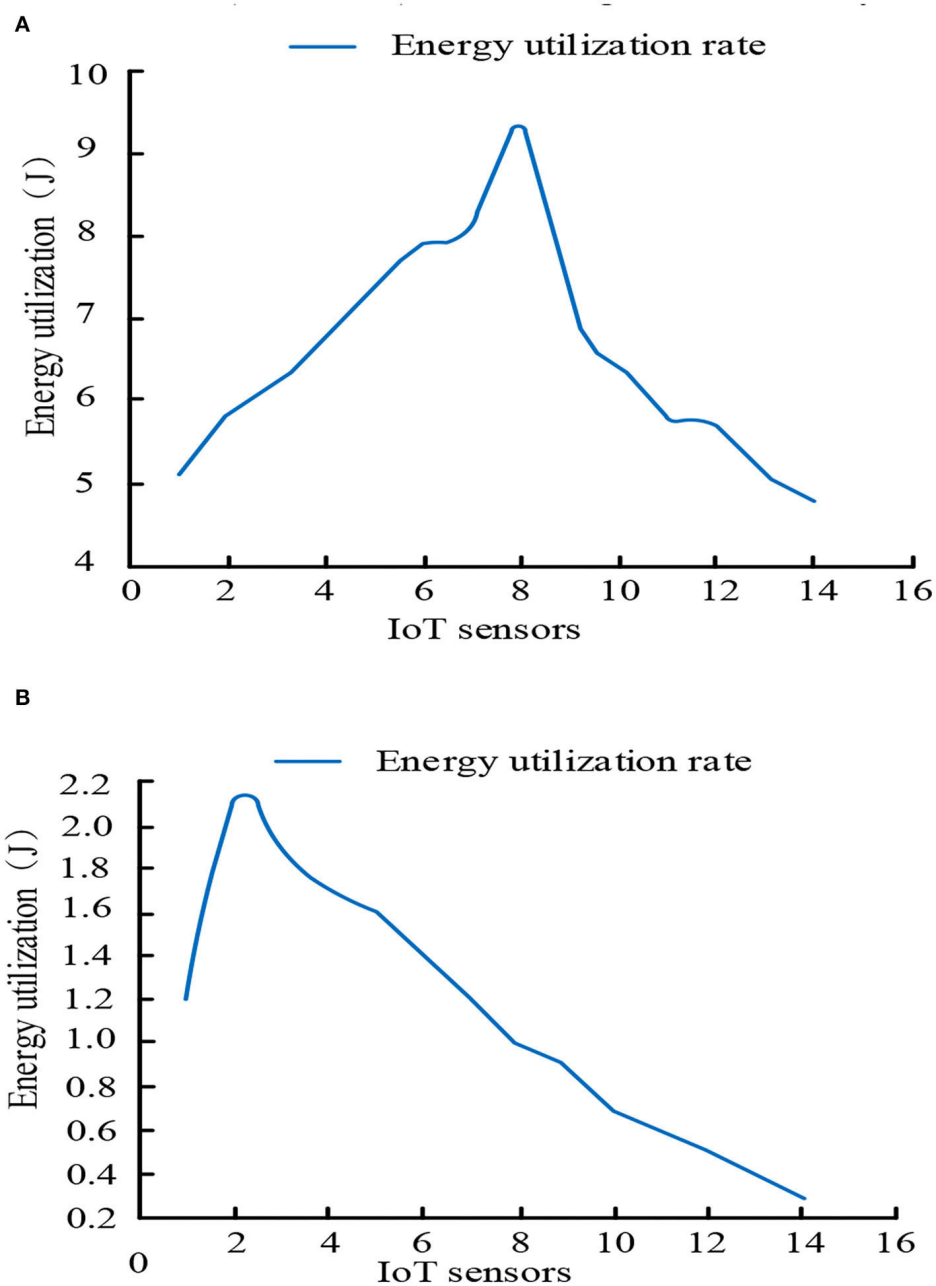
Comparative analysis of energy utilization between Im-HMS system and UCD system. **(A)** Energy utilization analysis of the existing UCD system. **(B)** Energy utilization analysis of the proposed Intelligent m-Health monitoring system (Im-HMS).

Figures 7A,B shows the energy utilization analysis of the existing system and the proposed intelligent mobile health monitoring system (Im-HMS), respectively. The energy utilization of each IoT device of the proposed intelligent mobile health monitoring system (Im-HMS) and existing systems is calculated. The results show that the system has the lowest energy consumption compared with existing UCD systems in IoT devices. The proposed system consumes 64% of the existing system's energy to monitor users.

The proposed intelligent mobile health monitoring system (Im-HMS) is designed and implemented. Simulation results such as accuracy, efficiency, energy utilization, end-to-end latency, and margin of error are analyzed. The results show that the proposed intelligent mobile health monitoring system (Im-HMS) outperforms existing UCD systems in all scenarios.

## Conclusion and findings

This paper presents key aspects and capabilities of the intelligent mobile health monitoring system (Im-HMS)—a breakthrough mobile health network that supports mobile and Fitbit technologies for digital exercise tracking and health training in CR customers. CR is an interdisciplinary lifestyle procedure with reliable data showing changes in several health outcomes. Im-HMS ensures routine CR-based services by increasing patient participation and performance on non-CR days. It establishes an alternative delivery model for CHD clients who are unable to join the center-based CR service to satisfy and motivate them. Im-HMS effectively integrates complex wearable and smartphone technologies in an economical and cost-effective manner by providing group therapy for real-time remote control, direct review, patient ownership, and more CHD autonomy. The general Im-HMS system definition can be transferred to other chronic diseases and diseases, obesity, asthma, and severe respiratory obstruction. This paper analyzes the performance of the proposed Im-HMS system and compares it with the UCD system in terms of accuracy, delay time, error range, efficiency, and energy utilization. The experimental results show that its performance is superior to that of the UCD system. The Im-HMS system proposed in this paper is of great significance for patient health monitoring in practical applications. It is worth noting that the model proposed in this paper still has shortcomings, and the model can be improved in future by integrating the advantages of deep learning technology in health monitoring.

## Data availability statement

The original contributions presented in the study are included in the article/supplementary material, further inquiries can be directed to the corresponding author.

## Author contributions

XL contributed to motivation, interpretation of methods, data analysis, results, provided draft, revised versions, and references. KY provided data and results, revisions and references, provided relevant concepts and small suggestions, and extracted conclusions and discussions. All authors contributed to the article and approved the submitted version.

## Funding

This study was supported by the Application and Promotion of Scientific Fitness, Xiyang County Health, and Sports Bureau.

## Conflict of interest

The authors declare that the research was conducted in the absence of any commercial or financial relationships that could be construed as a potential conflict of interest.

## Publisher's note

All claims expressed in this article are solely those of the authors and do not necessarily represent those of their affiliated organizations, or those of the publisher, the editors and the reviewers. Any product that may be evaluated in this article, or claim that may be made by its manufacturer, is not guaranteed or endorsed by the publisher.
